# Supplementary material for: Content-rich biological network constructed by mining PubMed abstracts
Source: BMC Bioinformatics. 2004 Oct 8;5:147. doi: 10.1186/1471-2105-5-147 (PMC528731; doi:10.1186/1471-2105-5-147)
Supplement: Additional File 2 — The original results of the above study (non-essential files are deleted to keep the file size under the limit set by BMC bioinformatics). [file 1471-2105-5-147-S2.bz2 › chilibotAdditionalFile2/dip05/546ID11043577E10899/html/AR_FLNA.html]

 


 **AR** and **FLNA** 
  
Found 1 abstracts in PubMed, retrieved 1.  
 

 What does Google say? 
 PDF only 
| .edu only 

---

**Interactive relationship** (e.g. stimulation, inhibition, etc)

**Neutral relationship**- Although full length  **FLNa**  was predominantly cytoplasmic, a C terminal 100 kDa fragment of  **FLNa**  colocalized with  **AR**  to the nucleus.  Ref: 12682292 Proc Natl Acad Sci U S A, 2003
- Filamin A  [ **FLNA** ]  fragment localizes to the nucleus to regulate androgen receptor  [ **AR** ]  and coactivator functions.  Ref: 12682292 Proc Natl Acad Sci U S A, 2003
- We have characterized a negative regulatory domain in the  **AR**  hinge region, which interacted with filamin A  [ **FLNA** ]   **FLNa** , an actin binding cytoskeletal protein.  Ref: 12682292 Proc Natl Acad Sci U S A, 2003
- **FLNa**  interfered with  **AR**  interdomain interactions and competed with the coactivator transcriptional intermediary factor 2 to specifically down regulate  **AR**  function.  Ref: 12682292 Proc Natl Acad Sci U S A, 2003

**Non-interactive relationship** (e.g. studied together, co-existance, homology, etc.)

- This naturally occurring  **FLNa**  fragment repressed  **AR**  transactivation and disrupted  **AR**  interdomain interactions and transcriptional intermediary factor 2 activated  **AR**  function in a manner reminiscent of full length  **FLNa** , raising the possibility that the inhibitory effects of cytoplasmic  **FLNa**  may be transduced through this fragment, which can localize to the nucleus and form part of the pre initiation complex.  Ref: 12682292 Proc Natl Acad Sci U S A, 2003
